# A Study of the North Water Polynya Ice Arch using Four Decades of Satellite Data

**DOI:** 10.1038/s41598-019-56780-6

**Published:** 2019-12-30

**Authors:** R. F. Vincent

**Affiliations:** Royal Military College of Canada, Department of Physics and Space Science, Kingston, Ontario, K7K 7B4 Canada

**Keywords:** Environmental sciences, Physical oceanography, Climate change

## Abstract

Polynyas are sections of the polar ocean that remain relatively ice-free during winter, imparting significant physical and biological impact on the region. The North Water polynya (NOW) situated between Ellesmere Island and Greenland is the largest recurring Arctic polynya. Historically, the NOW forms every season when Arctic Ocean floes moving southward through Nares Strait become congested and form an ice arch that defines the northern border of the polynya. This blockage usually forms during winter and breaks down in spring. It is conjectured that the polynya is maintained by latent heat of fusion from the continuous formation of new ice as floes are swept southward from the ice arch by wind and ocean currents. Analysis of four decades of satellite imagery indicates a growing instability in the location of the ice arch, challenging previous models of polynya maintenance. A linear trend of the data indicates the number of days of Nares Strait blockage has decreased 2.1 days/year between 1979 and 2019 with wide interannual variations. Prior to 2007, ice arches blocked Nares Strait an average of 177 days/year compared to 128 days/year since that time. The overall trend of reduced ice arch duration is a contributing factor to the dramatic loss of multiyear ice in the Arctic basin.

## Introduction

Polynyas are areas of the polar ocean that are covered by open-water and thin ice under climatic conditions that would normally dictate thick ice cover. These regions are generally classified as *latent* or *sensible heat* polynyas. For a latent heat polynya, ice is removed from the area by winds and/or ocean currents as quickly as it forms. Heat loss to the atmosphere is balanced by latent heat of fusion released by the continual formation of ice. For a sensible heat polynya, oceanic heat provided by the vertical mixing of warmer water from depth due to wind-induced upwelling, or through advection from ocean currents, prevents the formation of ice. Polynyas impart significant oceanographic, climatic and biological impact on the environment. They dominate the regional heat budget in winter with an ocean-to-atmosphere heat flux approximately two orders of magnitude higher than the surrounding pack ice^1^. The continuous formation of ice in polynyas leads to salt rejection and the subsequent formation of water masses and local currents. Interannual changes in polynya characteristics may contribute to the variability of the polar climate, reflecting large-scale climatic changes^2^. Polynyas constitute key habitats for seabirds and marine mammals^3–5^, serving as areas of high productivity and biodiversity^6,7^. As a result, polynyas are considered oceanographic ‘windows’ that allow an evaluation of the state of the polar marine ecosystem^8,9^.

**Figure 1:**
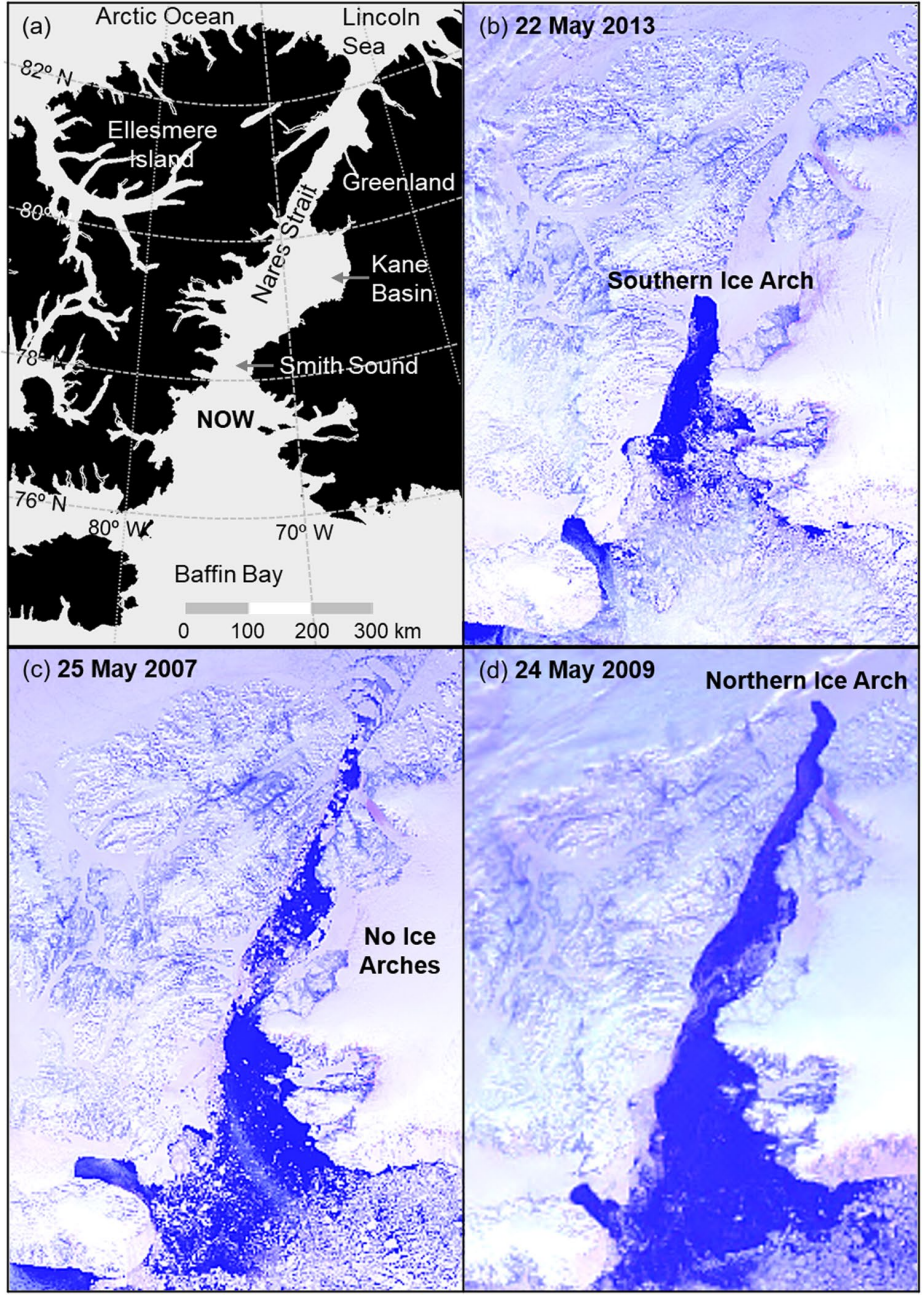
(**a**) A map of the North Water polynya (NOW) and surrounding area. (**b**) An ice arch at the narrow head of Smith Sound historically defines the northern boundary of the polynya, creating the iconic shape of the NOW. In recent years there have been unprecedented ice configurations in the NOW. (**c**) In 2007 the Smith Sound ice arch failed to consolidate for the first time on record. (**d**) In 2009 the Smith Sound ice arch again failed to form, but an anomalous ice arch at the northern point of Nares Strait dominated the region. (Images created with Harris Geospatial ENVI 5.3 software, http://www.harrisgeospatial.com. Satellite images are Advanced Very High Resolution Radiometer, 1.1 km resolution, UTM 18 map projection, Channels 1, 2, 4).

The North Water polynya (NOW) situated between Ellesmere Island and Greenland in northern Baffin Bay is the largest recurring polynya in the Canadian Arctic (Fig. 1a) and one of the most biologically productive marine areas in the Arctic^10–13^. Historically, pack ice transported southward from the Arctic Ocean through Nares Strait becomes congested and forms a blockage, or ice arch, across the narrow head of Smith Sound. The formation of ice arches is common in the narrow water passages of the Canadian Arctic Archipelgo (CAA)^14^ and this process is considered a critical aspect of the NOW’s formation and maintenance^2,8,15–20^. The Smith Sound ice arch usually forms in winter or early spring at which point ice is continuously swept southward from the blockage by prevailing winds and ocean currents^21–23^. The subsequent formation of new ice releases latent heat, which is an important mechanism for maintaining the polynya^24^. Other factors contributing to keeping the area relatively free of ice include strong tidal fluctuations^25^ and potential sensible heat influx through the upwelling of warm water near the Greenland coast^13,26^. While the Smith Sound ice arch sharply defines the northern limit of the polynya (Fig. 1b), the southern boundary is characterized by diffuse pack ice in northern Baffin Bay. The NOW is not fully ice-free in winter^1^ with an estimated 50% of Smith Sound covered with ice greater than 30 cm thick in February and March^27^. Since the mid-1990s there has been an expansion of open water in the polynya during winter and greater occurrence of thinner ice^28^. Generally, the winter pack ice begins to disperse in early spring and the polynya expands southward from Smith Sound, reaching a maximum water area of 80,000 km^2^ in July. The Smith Sound ice arch usually breaks down in June or July at which point the polynya is indiscernible as ice floes from the Arctic Ocean enter the area through Nares Strait.

The western part of the NOW is characterized by cold outflow (−0.6 °C) from Nares Strait, with a mean surface current speed ranging from 10 to 15 cm s^−1^ ^24^. The strongest tides in the Canadian Arctic occur north of the NOW in Kane Basin with modeled amplitudes reaching 135 cm^29^, which can reverse the Nares Strait current and significantly change the ice dynamics on a diurnal basis^25^. On the eastern side, the relatively warm West Greenland Current (2.0 °C) flows northward along the Greenland coast with a surface current speed of 3 to 5 cm s^−1^ until crossing the southern portion of the NOW to join the Nares Strait outflow and form the southward moving Baffin Current^24^. Nares Strait, which averages 30 to 50 km in width and extends 500 km from the Arctic Ocean to the NOW, transports an annual average area of 40,000 km^2^ of ice from the Arctic Basin into Baffin Bay^30^ with multiyear ice contributing a significant proportion to this amount^31^. Multiyear ice with drafts exceeding 5 m constitute up to 16% of the observed sea ice traversing Nares Strait, draining the last reservoir of old multiyear ice in the Lincoln Sea north of Ellesmere Island^32^. The formation and duration of ice arches in the Nares Strait system impacts the rate of ice loss from this reservoir, which is part of a region that climate models predict will be the last to lose perennial ice cover in the Arctic Ocean known as the Last Ice Area^33^.

**Figure 2:**
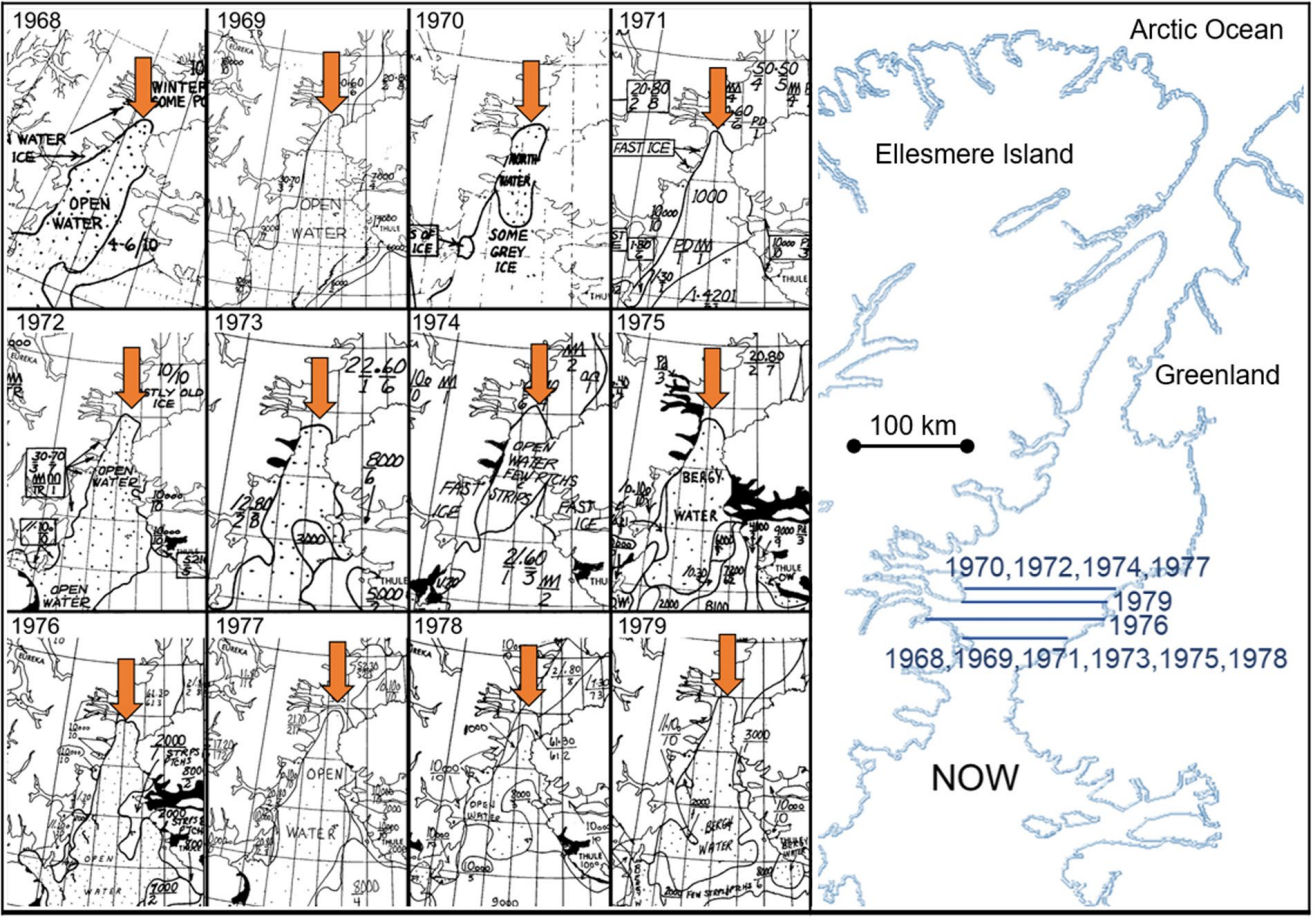
Government of Canada archival ice charts illustrate the stability of the Smith Sound ice arch. On the left side, the ice arch is indicated with red arrows for the month of June between 1968 and 1979. The right side is a plot of the northern most point of the ice arch in accordance with the charts. (Ice charts are available at https://iceweb1.cis.ec.gc.ca/Archive/page1.html?lang = en).

The establishment of bird colonies and human presence in the Thule, Greenland region indicate that the NOW was established about 4,500 years ago^34^. The diverse ecological system of the NOW made it possible for the prehistoric Thule Inuit to settle and their descendants survive in the region since 1250 AD^34,35^. Historical references to the NOW extend back to the voyages of William Baffin from 1612 to 1618 in which he discovered a great “open sea” south of what is now known as Kane Basin^36^. In 1861 Isaac Hayes, an American Arctic explorer, wintered at the narrowest section of Smith Sound where his sailing progress was stopped by the ice arch^37^. Air ice reconnaissance in the 1950s and 1960s allowed the Smith Sound ice arch to be drawn on charts with some precision^38^. The first scientific journal to feature the NOW emphasized that the most striking aspect of the polynya was the “extreme” stability of the “ice bridge” that defines the northern border^39^. The consistency of the Smith Sound ice arch from 1968 to 1979 is illustrated by ice charts created during this era in which it averages a seasonal latitude of 79.2 ± 0.5°N. (Fig. 2).

Beginning in October 1978, the availability of consistent satellite imagery of the Arctic in visible and thermal infrared (IR) wavelengths permitted accurate tracking of the annual evolution of the NOW ice arch. In recent years, satellite data has revealed atypical ice configurations in the NOW. In 2007 the Smith Sound ice arch failed to consolidate (Fig. 1c), which allowed a record area (87,000 km^2^) and volume (254 km^3^) of Arctic Ocean ice to pass through Nares Strait in a one-year period^31^. In 2009 the Smith Sound ice arch failed to form again, but a different ice arch at the northern terminus of Nares Strait (Fig. 1d) prevented ice floes from entering Nares Strait between 08 January and 12 July. Northern ice arches are relatively common in Nares Strait, but they historically precede the formation of the Smith Sound ice arch by 10 to 30 days^31^. The anomalous 2009 northern ice arch persisted for 184 days and caused historically low ice cover in the NOW, leading to unprecedented sea surface temperatures in the region^40^ that caused early phytoplankton blooms^13^. The Smith Sound ice arch did not consolidate in 2010, 2017 and 2019, although a northern ice arch formed for a short period in each of these years.

The changing ice arch dynamics of the NOW impacts the state of multiyear ice in the Arctic Ocean as well as the delicate ecosystem of the region and the people who have come to rely on this Arctic oasis. This research analyzes the NOW ice arches using visible and thermal IR satellite imagery from 1979 to 2019. The location and duration of ice arches over the past forty years are investigated and trends in the data examined.

## Methods

Advanced Very High Resolution Radiometry (AVHRR) imagery was chosen for this study owing to its long heritage, continuity, availability and ease of processing. The AVHRR sensor is capable of imaging the Earth in visible and thermal IR wavelengths with a spatial resolution of 1.1 km at nadir. The first AVHRR was launched in October 1978 on the Television Infrared Observation Satellite - Next Generation (TIROS-N), which was put into service by the National Oceanic and Atmospheric Administration (NOAA). The follow-on to TIROS-N was NOAA-6 in 1979, at which point the NOAA spacecraft took on numerical designations upon reaching operational status^41^. NOAA satellites equipped with the AVHRR sensor have been monitoring the Earth continuously since 1978 with the latest version (NOAA-19) launched in 2009. Between 2006 and 2018 the European Space Agency launched three MetOp satellites (A, B and C) that also carry an AVHRR. Eighteen satellites in total have operated an AVHRR, six of which are in operation in 2019. The latest NOAA polar orbiting satellite (NOAA-20) launched in 2017 replaced the AVHRR with the Visible Infrared Imaging Radiometer Suite developed from NASA’s Moderate Resolution Imaging Spectroradiometer flown on the Aqua and Terra satellites. Table 1 lists the parameters and variations of the AVHRR as well as the timeline of satellites that have flown with the sensor.

**Table 1:** Parameters and variations of the Advanced Very High Resolution sensor and timeline for satellites carrying the sensor^41^.

AVHRR1/2/3 Parameters					
Channel	Nadir Resolution	Wavelength (um)	Channel	Nadir Resolution	Wavelength (um)
1	1.1 km	0.58–0.68	3B	1.1 km	3.55–3.93
2	1.1 km	0.725–1.00	4	1.1 km	10.30–11.30
3 A	1.1 km	1.58–1.64	5	1.1 km	11.50–12.50
**AVHRR Equipped Satellites**					
**Satellite**	**Launched**	**End of Mission**	**Satellite**	**Launched**	**End of Mission**
TIROS-N	Oct 1978	Feb 1981	NOAA-14	Dec 1994	Oct 2001
NOAA-6	Jun 1979	Nov 1986	NOAA-15**	May 1998	—
NOAA-7	Jun 1981	Jun 1986	NOAA-16	Sep 2000	Jun 2014
NOAA-8	Mar 1983	Oct 1985	NOAA-17***	Jun 2002	Apr 2013
NOAA-9	Mar 1984	Nov 1995	NOAA-18	May 2005	—
NOAA-10	Sep 1986	Oct 2000	MetOp -A	Oct 2006	—
NOAA-11	Sep 1988	Sep 1994	NOAA-19	Feb 2009	—
NOAA-12	May 1991	Aug 2007	MetOp -B	Sep 2012	—
NOAA-13*	Aug 1993	Aug 1993	MetOp -C	Nov 2018	—

AVHRR/1: 1,2,3b,4 carried on TIROS-N to NOAA-7 satellites.

AVHRR/2: Channels 1,2,3b,4,5 carried on NOAA-8 to NOAA-14 satellites.

AVHRR/3: Channels 1,2,3a,3b,4,5 carried on NOAA-15 to NOAA-19 and MetOp series satellites.

*NOAA-13 experienced a power failure two weeks after launch.

**NOAA-15 experienced a problem with the AVHRR sensor Jul 2019.

***NOAA-17 AVHRR sensor turned off Apr 2010.

All satellites equipped with an AVHRR are in low Earth polar orbits that pass over the Arctic approximately 14 times per day. The sensor has a swath of approximately 2,900 km, allowing the NOW region to be imaged five to seven passes in a 24-hour period. The 1.1 km spatial resolution at nadir degrades to about 8 km toward the edge of the swath but is still sufficient to define the ice arch structures. File formats offering the best spatial resolution are High Resolution Picture Transmission for the NOAA series and Full Resolution Area Coverage for the MetOp series. For earlier satellite images only Global Area Coverage format was available offering 4 km resolution at nadir. The reduced spatial resolution was found to be adequate for analyzing ice arch structures. All AVHRR data utilized in this study was retrieved on-line from NOAA’s Comprehensive Large Array-data Stewardship System for various AVHRR-equipped satellites from 1978 to 2019 (https://www.bou.class.noaa.gov/saa/products/welcome).

**Figure 3:**
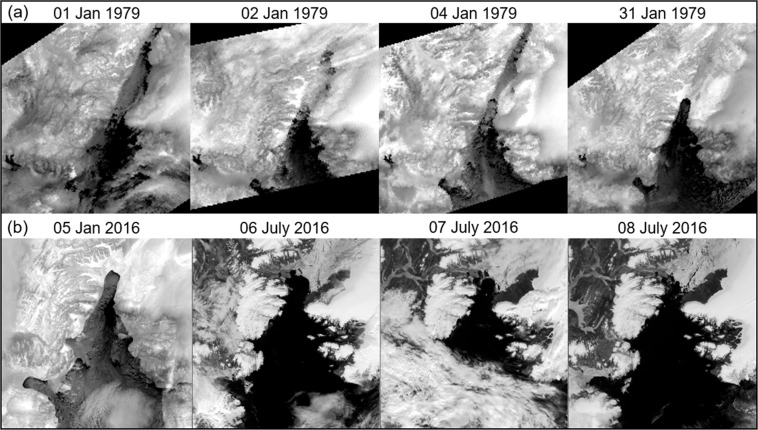
An example of the formation and breakdown of the southern ice arch using Advanced Very High Resolution Radiometer thermal infrared imagery. (**a**) On 04 January 1979 the characteristic shape of the southern ice arch is established for the season as verified in the 31 January 1979 image. (**b**) The break down of the southern ice in 2016 using visible wavelengths. The characteristic shape for the season as seen of 05 January 2016 collapses on 07 July 2016. (Images created with Harris Geospatial ENVI 5.3 software, http://www.harrisgeospatial.com).

The time and location of the formation and break down of the southern ice arch (Smith Sound) and northern ice arch (head of Nares Strait) was determined for each season from 1979 to 2019 by visibly examining individual AVHRR images for each day. The term ‘season’ is used since the formation of an ice arch sometimes occurred in the previous calendar year of the ice arch break down. The darkness of Arctic winter requires the use of thermal IR imagery to determine ice arch formation, while visible wavelengths are superior for observing the break down of these structures. An ice arch was determined to be fully formed when it achieved the characteristic shape for the season, whereas the break down date was signified by the initial collapse of the arch structure. Figure 3 is an example of ice arch formation and break down. The unique ice arch shape for each year is maintained throughout the season, which helps to identify the presence of the formation under thin cloud or amidst thin ice. There is commonly extensive cloud cover in the Arctic maritime environment during the warmer months^42,43^, which made satellite observation of the ice arch break down difficult for some seasons. However, the high number of satellite passes per day allowed the date of the event to be confidently assessed to within two days. The development of ice arches in winter was relatively straight forward owing to the reduced cloud cover and excellent contrast between ice and water provided by the thermal IR imagery. Since the assessment of ice arch formation and break down may differ between researchers and clouds can mask the exact day of formation, a ± 2-day uncertainty is attached to these timings to account for potential discrepancies. Incidences in which ice arches formed for a few days but did not completely solidify are not included in the results. The goal was to determine the continuous number of days that Nares Strait was blocked by ice arches for each season and the time frame of the blockage. The latitude of the ice arch was designated by the most northern point of the structure, which usually occurs at the center. Although ‘arch’ is the generic term for the structure, there are years when it goes straight across the channel like an ‘ice bridge’.

## Results

**Figure 4:**
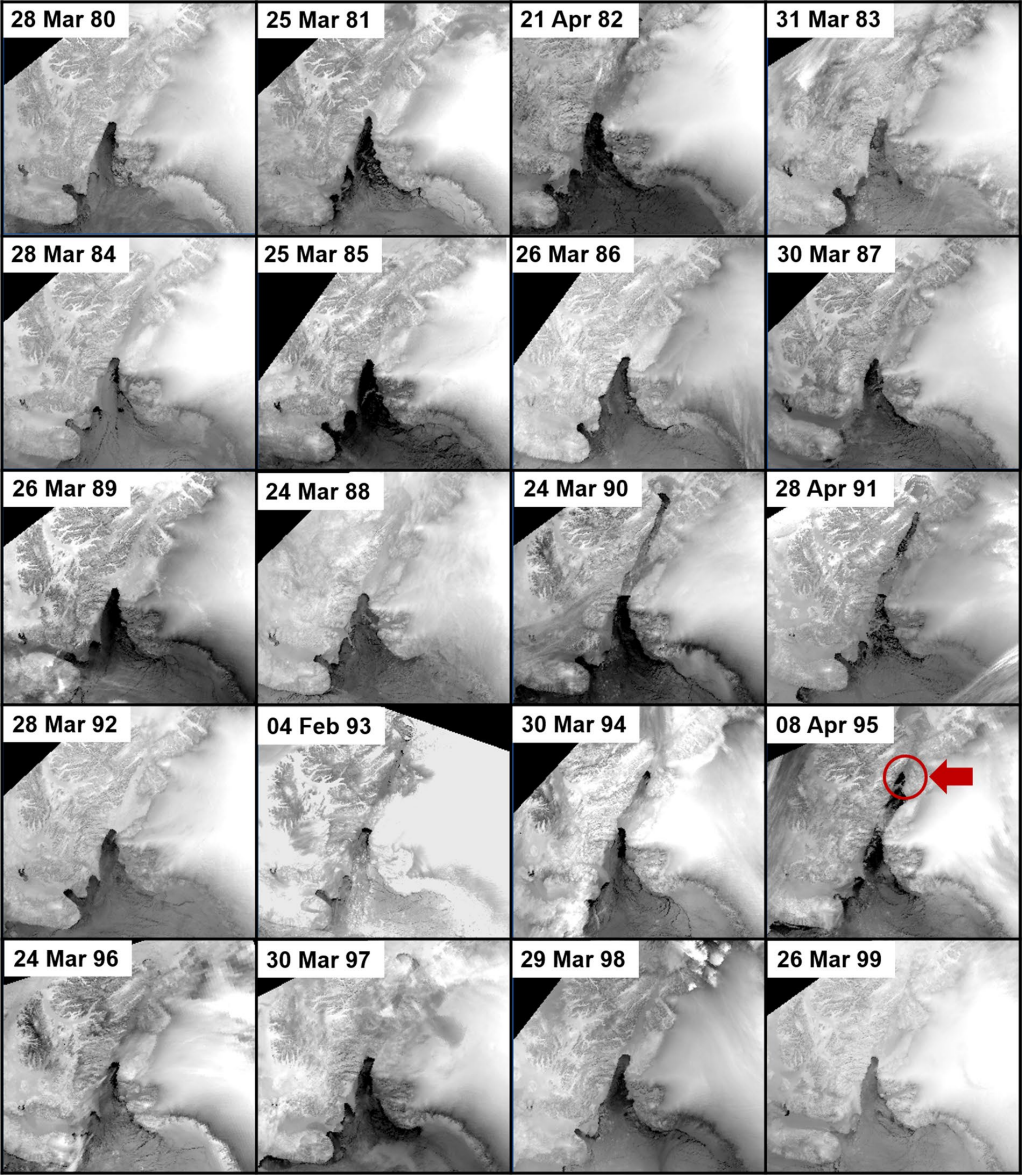
Thermal infrared Advanced Very High Resolution Radiometer images of the North Water polynya from 1980 to 1999. The position of the ice arch is consistent except for 1995 when an ice arch formed at the mid point of Nares Strait (see circled area). The shape of the ice arch takes on different variations from one year to another. (Images created with Harris Geospatial ENVI 5.3 software, http://www.harrisgeospatial.com).

**Figure 5:**
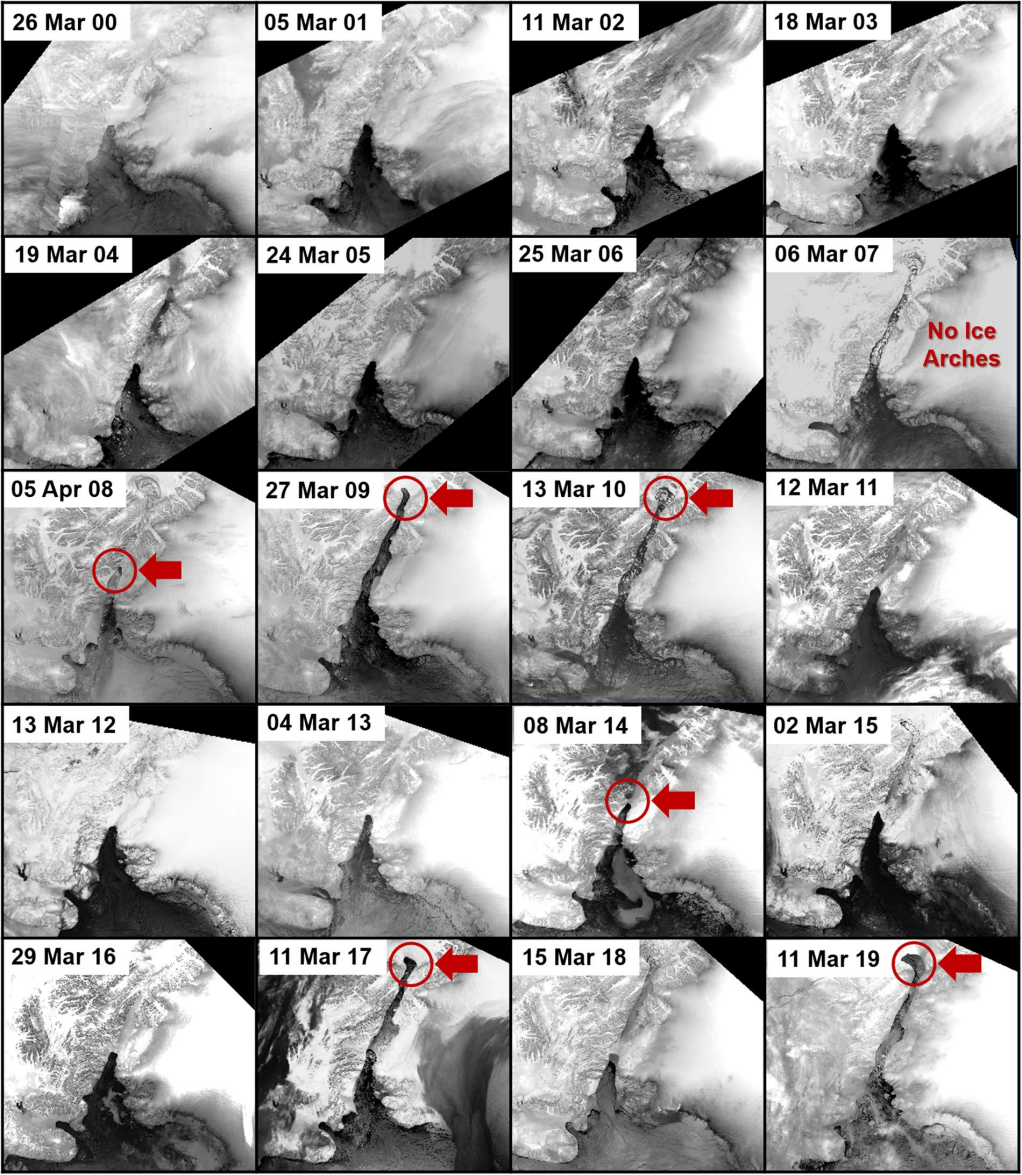
Thermal infrared Advanced Very High Resolution Radiometer images of the North Water Polynya from 2000 to 2019. Beginning in 2007 seven different ice arch anomalies occurred as indicated in red. In 2007 no ice arches consolidated in Nares Strait for the first time in recorded history. In 2009 only the northern ice arch formed, which was another first for the region. Northern ice arches predominated again in 2010, 2017 and 2019 while anomalous southern ice arches occurred in 2008 and 2014. (Images created with Harris Geospatial ENVI 5.3 software, http://www.harrisgeospatial.com).

Figures 4 and 5 show the state of the dominant ice arches for the NOW from 1980 to 1999 and 2000 to 2019 respectively. Table 2 gives the date of formation and break down of the northern and southern ice arch for each year and the number of days of continuous ice arch blockage of Nares Strait. The data compares relatively well with Kwok *et al*. (2010) who used satellite synthetic aperture radar data to analyze ice flux though Nares Strait from 1997 to 2009. Discrepancies in the two datasets may be attributed to the sensor used, methods, temporal sampling and the different overall aim of the studies.

**Table 2:** Dates for ice arch formation and break down from 1979 to 2019. Arch days indicate the number of days that Nares Strait was continuously blocked by ice arches.

Sea-son	N. Arch Form	S. Arch Form	Break-down	Arch Days	Sea-son	N. Arch Form	S. Arch Form	Break-down	Arch Days
2019	16 Feb	—	20 Mar	32	1999	—	25 Jan	10 Jul	166
2018	—	04 Mar	30 Jun	118	1998	09 Jan	07 Mar	22 Jun	164
2017	23 Jan	—	11 May	108	1997	04 Mar	13 Mar	22 Jul	140
2016	—	05 Dec 15	07 Jul	215	1996	—	01 Feb	28 Jul	178
2015	—	14 Feb	16 Jul	152	1995	—	16 Mar	19 May	64
2014	09 Dec 13	15 Jan	10 Jul	213	1994	23 Dec 93	25 Mar	14 Jun	173
2013	—	08 Nov 12	10 Jul	243	1993	—	27 Jan	06 Feb	10
2012	—	06 Dec 11	29 Jun	206	1992	—	05 Dec 91	21 Jul	229
2011		31 Jan	18 Jun	138	1991	—	05 Apr	12 Jul	98
2010	15 Mar	—	16 Apr	32	1990	—	20 Mar	3 May	44
2009	09 Jan	—	12 Jul	184	1989	—	09 Dec	27 Jul	230
2008	—	01 Apr	07 Jun	67	1988	06 Dec 87	15 Dec 87	13 Jul	220
2007	—	—	—	0	1987	—	20 Jan	16 Jul	177
2006	—	08 Feb	30 Jun	142	1986	—	24 Dec 85	12 Jul	200
2005	03 Dec 04	06 Dec 04	15 Jul	224	1985	—	01 Dec 84	12 Jul	223
2004	14 Feb	09 Mar	02 Jul	139	1984	14 Nov 83	15 Feb	21 Jul	250
2003	—	25 Feb	01 Jul	126	1983	27 Jan	01 Feb	11 Jul	165
2002	—	10 Dec 01	01 Jul	203	1982	—	06 Apr	05 Jul	90
2001	03 Jan	11 Jan	12 Jul	190	1981	20 Nov 80	04 Dec 80	28 Jul	250
2000	14 Oct 99	26 Oct 99	11 Jul	271	1980	—	11 Nov 79	17 Sep	310
**Note**: ± 2 days for dates, ± 4 for arch days					1979*	05 Nov 78	05 Nov 78 04 Jan	02 Aug	270+
**Average 1979 to 2019** **Standard Deviation (Days)**						**01 Jan** **41.9**	**20 Jan** **47.7**	**28 Jun** **38.8**	**161** **76.7**

*For the 1979 season an initial southern ice arch was observed for 05 Nov 1978. Earlier TIROS-N imagery is not available to determine the date the ice arch formed. This initial ice arch broke up on 28 Nov 1978, but Nares Strait remained blocked by a northern ice arch. A second southern ice arch consolidated on 04 Jan 1979.

The following is a decadal and overall summary of the data in this study.

### 1980 to 1989

**Figure 6:**
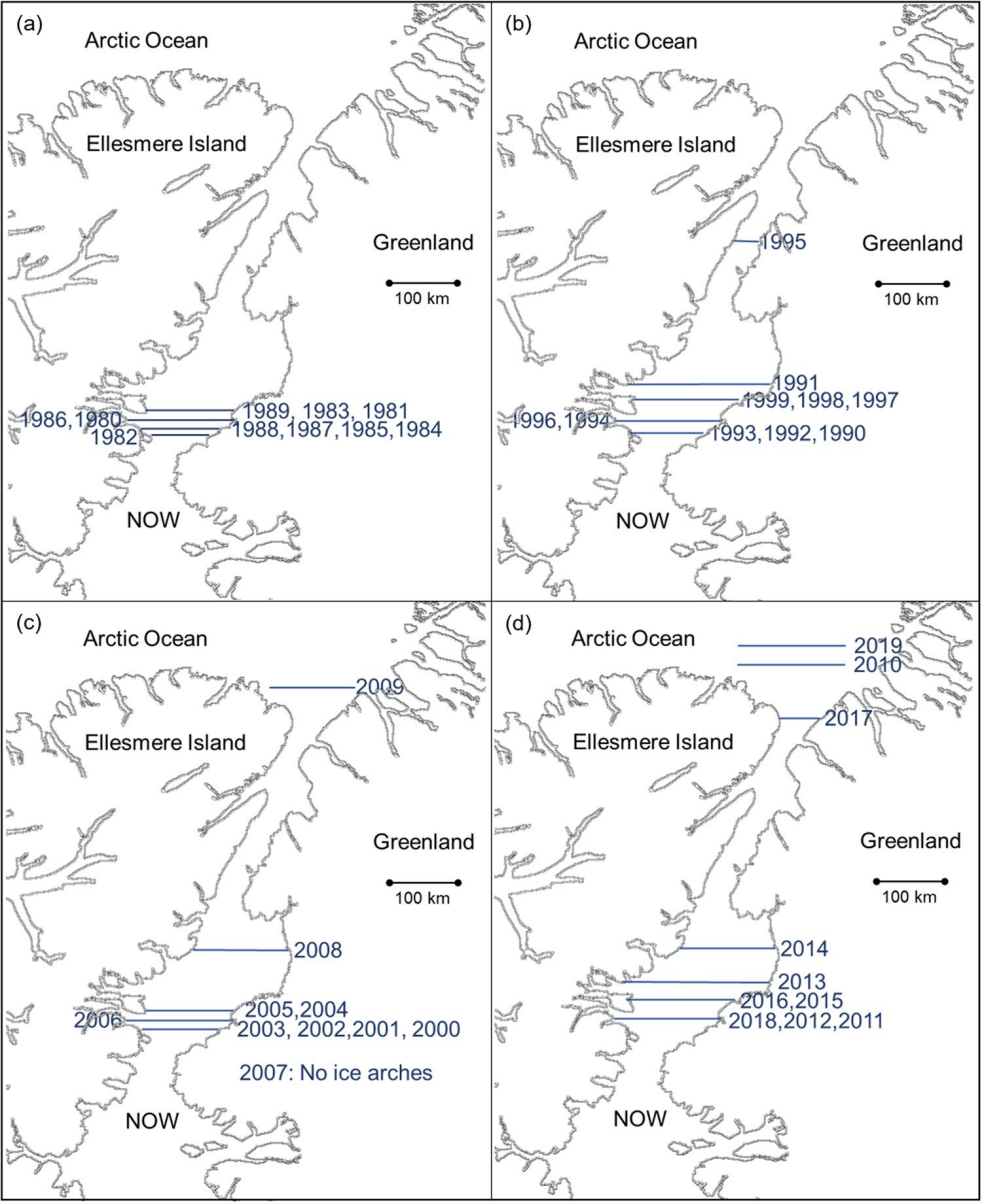
Decadal location of the most northern point of ice arches in Nares Strait for (**a**) 1980s (**b**) 1990s (**c**) 2000s and (**d**) 2010s. The southern ice arches are shown except for the years when only a northern ice arch formed. The consistent location of the southern ice arch noted in the 1970s (Fig. 2) and 1980s begins to change in the 1990’s. From 2007 to 2019 the periodic non-formation of the southern ice arch and the emergence of predominant northern ice arches emphasize the transformation of the typical ice configuration of the North Water polynya.

During the 1980s the southern ice arch formed each year in a confined region just north of Smith Sound with an average latitude of 78.9 ± 0.2° (Fig. 6a) which is similar to the pattern shown by ice charts between 1968 and 1979 (Fig. 2). The northern ice arch formation preceded the southern ice arch on four occasions (1981, 1983, 1984, 1988). Historically, in years that the northern ice arch forms the southern ice arch consolidates within 30 days, which is followed by the eventual freezing of Nares Strait. In 1984 the northern ice arch preceded the southern ice arch by a record 93 days. The average continuous ice arch blockage of Nares Strait for the decade was 221.5 days per season, ranging between 90 days (1982) and 310 days (1980).

### 1990 to 1999

The 1990s experienced the lowest decadal continuous ice arch blockage of Nares Strait, averaging 126.5 days per season, with a range of 10 days (1993) to 229 days (1992). The location of the southern ice arch is more variable than observed in previous decades (Fig. 6b) and is highlighted by an ice arch in 1995 that formed at approximately the mid point of Nares Strait 240 km north of the normal position. The edge of this anomalous ice arch retreated northward during May before collapse. The northern ice arch formed first in three seasons during the decade (1994, 1997, 1998) with the 1994 formation preceding the southern ice arch by 92 days. The 1990s are highlighted by the first known significant variation in the ice arch location and featured a year (1993) in which Nares Strait was only blocked for 10 days by a fully consolidated ice arch. The 1990s also marked a sudden expansion in polynya size during winter (November to March), which appears to be linked to warmer temperatures brought about by a transition to a prevailing more negative phase of the North Atlantic Oscillation^28^.

### 2000 to 2009

Between 2000 and 2006 the southern ice arch showed great stability, forming at an average latitude of 78.8 ± 0.1°N (Fig. 6c). The northern ice arch formed first four times during this time frame (2000, 2001, 2004, 2006) preceding the southern ice arch formation by 3 to 24 days. During the 2007 season no ice arches solidified in Nares Strait for the first time in recorded history and contributed to record low ice coverage in the Arctic Ocean that year^31^. In 2008 the southern ice formed in the northern section of Kane Basin, approximately 100 km north of the normal position. This was followed by the formation of only the northern arch in 2009. This ice configuration, which lasted for 184 days, had never been observed before and led to a significant warm water anomaly in Baffin Bay in July primarily as a result of low ice coverage in the region^40^. The average continuous blockage of Nares Strait for the decade was 154.6 days per season, ranging from 0 days (2007) to 224 days (2005).

### 2010 to 2019

The striking aspect of the 2010s is the wide spatial distribution of the ice arches (Fig. 6d). For three seasons only the northern ice arch fully consolidated (2010, 2017, 2019) while a southern ice arch in 2014 formed in the northern section of Kane Basin. The northern ice arch formed prior to the southern ice arch only once (2011) with a span of 84 days between the two formations. The average continuous blockage of Nares Strait for the decade was 141.7 days per season, ranging from 32 days (2010, 2019) to 243 days (2019). It should be noted that the collapse of an ice arch does not necessarily lead to the immediate free flow of ice through Nares Strait. As an example, in 2018 the southern ice arch broke down on 30 Jun, but the resultant collapse led to an ice plug in Kane Basin that impeded southward ice floe movement until the beginning of August.

### Overall 1979 to 2019

**Figure 7:**
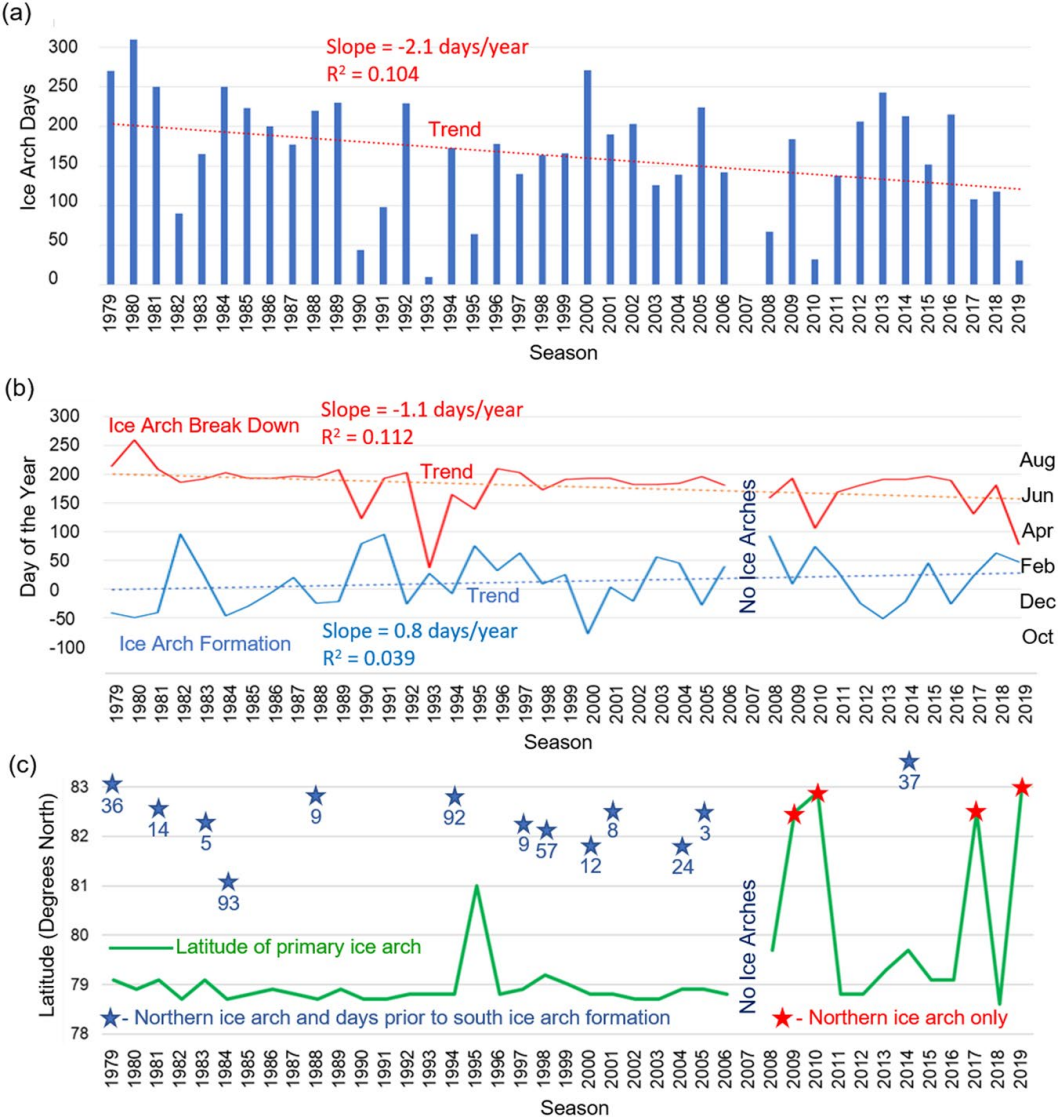
(**a**) The number of continuous ice arch days is highly variable but there is an overall decrease of 2.1 days/year between 1979 and 2019. (**b**) The date of ice arch formation is more variable than breakdown. The overall trend is for later formation and earlier break down. (**c**) The latitude of the primary ice arch is relatively constant until 2007 at which point a number of anomalies occur. The blue stars indicate seasons in which the northern ice arch preceded the southern ice arch and the delay in days between the formations. The red stars indicate seasons in which only the northern ice arch formed.

Figure 7a shows the number of days ice arches continuously blocked Nares Strait for each season from 1979 to 2019. There is considerable variation between seasons, but a linear trend reveals that the number of ice arch days is decreasing by 2.1 days/year. The variability of the data leads to a low R^2^ value (0.104), which means that despite the downward trend of ice arch days it is statistically difficult to predict what will happen in any particular year in the future. The average continuous blockage of Nares Strait between 1979 and 2019 is 161 days per season. Prior to 2007 this value was 177 days compared to 128 days since 2007, which represents the beginning of an era of frequent ice arch anomalies.

The time of year for initial ice arch formation and the date of ice arch break down is shown in Fig. 7b. The data reveals high interannual variability in the formation of the ice arch with more consistency shown in the break down of the structure. A linear trend indicates that the ice arch is forming 0.8 days/year later and breaking down 1.1 days/year earlier. The R^2^ values for these trends are 0.039 and 0.112 respectively, indicating a low statistical significance for predicting the future as a result of the high variability of the data.

Figure 7c summarizes the location of both the northern and southern ice arches. Except for 1995 when an ice arch formed part way up Nares Strait, the location of the southern ice arch shows good consistency from 1976 to 2006. After the 2007 season in which no ice aches formed there is high variability in the location of ice arches, including four seasons of only a northern ice arch. There are 13 years in which a northern ice arch consolidated prior to the southern ice arch formation. The delay between the two ice arches averages 30.7 days, ranging from 3 to 93 days, with the location of the northern ice arch varying from 81.1° N to 83.5° N. The data suggests a temporal relationship between the northern and southern ice arch formation when both exist but the complicated nature of arching, which is a function of locally complex ocean and meteorological conditions, makes it difficult to determine a precise geophysical connection^31^.

## Discussion

Literature on the maintenance of the NOW indicates that the formation of the southern ice arch is a critical aspect of the polynya’s existence, but this does not appear to be the case. Since 2007 there have been five seasons when the southern ice arch did not fully consolidate, but the NOW has remained relatively ice free during all these years. Indeed, during the 2007 season when no ice arches consolidated in Nares Strait, the NOW reached record levels of winter polynya size^28^. The data suggests that other factors contributing to polynya maintenance play a more dominant role than the presence of the southern ice arch. For example, orographically channeled winds through Nares Strait accelerate through Smith Sound and lead to increased ice advection^44,45^. Additionally, the diurnal divergence and convergence of the pack ice in the NOW as a result of tides allow the escape of oceanic heat with the creation of leads in the ice^25^ while the formation of new ice during quiet tidal periods supply latent heat of fusion. Tides may also play a role in ice arch formation. For instance, during the consolidation of the 2009 northern ice arch, satellite data in this study showed a northward movement of floes in northern Nares Strait.

The emerging prominence of the northern ice arch since 2009 is an indication of changing ice dynamics in the region. Historically, for years when both ice arches formed the southern ice arch would consolidate within approximately 30 days of the northern ice arch, which coincides with the amount of time it takes for a floe to traverse the length of Nares Strait^40,46–48^. Delays of up to 93 days for the southern ice arch formation were observed prior to 2009, but eventually Nares Strait would freeze completely once the southern ice arch formed. This leads to the conclusion that the freezing of Nares Strait is linked to the formation of the southern ice arch.

The amount of Arctic Ocean ice loss during any particular year is complex, depending on the removal of ice by wind, ocean currents and *in situ* melting. These driving factors are affected by both large-scale and regional meteorological conditions. The trend toward younger, thinner ice in the Arctic basin makes it more vulnerable to melting as well as break up and export out of the region^49^. Arctic ice is generally transported southward through Fram Strait, the CAA and Nares Strait. The mean annual outflow through Fram Strait is 706,000 km^2^ ^50^, which is significantly higher than yearly averages for Nares Strait (40,000 km^2^)^30^ and the CAA (23,000 km^2^)^51^. However, unlike Fram Strait the ice transported through Nares Strait is dominated by thick multiyear ice from north of Ellesmere Island and Greenland^52^. The Last Ice Area is losing ice mass at double the rate of the Arctic Ocean as a whole, which is augmented by reduced ice arch blockages in Nares Strait^33^. The ice arches have been observed to contain thick multiyear ice held together with thin younger ice^31^ and it is suggested that ice arches in the region are less stable in the absence of thick ice^52^. Consequently, the observed temporal and spatial variability of the Nares Strait ice arches may be a function of the thinning and depletion of the ice reservoir north of Ellesmere Island.

Arctic amplification is the observation that near surface air temperatures are increasing more rapidly in the Arctic than the global average^53,54^ leading to a significant diminishment of ice in the Arctic Ocean over the past four decades. Physically and biologically, polynyas are considered a model for scientist to predict the Arctic system response to climate change^9^. After many years of stability, the iconic shape of the NOW as defined by the southern ice arch is no longer a certainty, reflecting the significant changes taking place in the Arctic icescape as a result of Arctic amplification. In 2009 the northern ice arch resulted in a virtually ice-free Baffin Bay in July, which led to unprecedented summertime sea surface temperatures largely as a result of decreased albedo and 24-hour solar insolation, affording insight to a seasonally ice-free Arctic Ocean^40^. Increased energy imparted to the ocean can lead to delayed winter freeze-up ^9,55^, while the additional sensible heat in the upper ocean results in enhanced heat transfer that may result in higher regional surface air temperatures^56^. The variability in the NOW ice arches since 2007 is a response to the warming climate. Continuing trends of anomalous ice arch location and diminishing duration will have an impact on the regional ecosystem and the storage of multiyear ice in the Last Ice Area.

## Data Availability

Satellite Advanced Very High Resolution (AVHRR) data is available on-line at the National Oceanic and Atmospheric Administration (NOAA) Comprehensive Large Array-data Stewardship System (CLASS)(https://www.bou.class.noaa.gov/saa/products/welcome). Archival ice charts are available on-line at the Government of Canada ice archive (https://iceweb1.cis.ec.gc.ca/Archive/page1.html?lang = en).

## References

[1] Smith S, Muench R, Pease C (1990). Polynyas and leads: An overview of physical processes and environment. Journal of Geophysical Research.

[2] Mysak L, Huang F (1992). A Latent-and Sensible-Heat Polynya Model for the North Water, Northern Baffin Bay. Journal of Physical Oceanography.

[3] Wallace, R. Polynyas in the Canadian Arctic, edited by Ian Stirling and Holly Cleator. *ARCTIC* 35, (1982).

[4] Karnovsky N, Hunt G (2002). Estimation of carbon flux to dovekies (Alle alle) in the North Water. Deep Sea Research Part II: Topical Studies in Oceanography.

[5] Heide-Jørgensen M (2012). The Significance of the North Water Polynya to Arctic Top Predators. AMBIO.

[6] Marchese C (2015). Biodiversity hotspots: A shortcut for a more complicated concept. Global Ecology and Conservation.

[7] Kalenitchenko, D., Joli, N., Potvin, M., Tremblay, J. & Lovejoy, C. Biodiversity and Species Change in the Arctic Ocean: A View Through the Lens of Nares Strait. *Frontiers in Marine Science* 6, (2019).

[8] Smith, W. O. & Barber, D. G. (Eds.) *Polynyas Windows to the World*. Elsevier Oceanography series, **74**, 458 pp (Amsterdam: Elsevier, 2007).

[9] Barber, D., Marsden, R. & Minnett, P. Preface: The international North Water (NOW) polynya study. *Atmosphere-Ocean* 39 (2001).

[10] Deming J, Fortier L, Fukuchi M (2002). The International North Water Polynya Study (NOW): a brief overview. Deep Sea Research Part II: Topical Studies in Oceanography.

[11] Klein B (2002). Phytoplankton biomass, production and potential export in the North Water. Deep Sea Research Part II: Topical Studies in Oceanography.

[12] Odate T (2002). Temporal and spatial patterns in the surface-water biomass of phytoplankton in the North Water. Deep Sea Research Part II: Topical Studies in Oceanography.

[13] Marchese C (2017). Changes in phytoplankton bloom phenology over the North Water (NOW) polynya: a response to changing environmental conditions. Polar Biology.

[14] Hibler W, Hutchings J, Ip C (2006). Sea-ice arching and multiple flow States of Arctic pack ice. Annals of Glaciology.

[15] Darby M, Willmott A, Mysak L (1994). A Nonlinear Steady-State Model of the North Water Polynya, Baffin Bay. Journal of Physical Oceanography.

[16] Biggs N, Willmott A (2001). A steady-state coupled ocean-polynya flux model of the North Water, Baffin Bay. Geophysical & Astrophysical Fluid Dynamics.

[17] Barber D, Hanesiak J, Chan W, Piwowar J (2001). Sea‐ice and meteorological conditions in Northern Baffin Bay and the North Water polynya between 1979 and 1996. Atmosphere-Ocean.

[18] Ingram R, Bâcle J, Barber D, Gratton Y, Melling H (2002). An overview of physical processes in the North Water. Deep Sea Research Part II: Topical Studies in Oceanography.

[19] Yao T, Tang C (2003). The formation and maintenance of the North Water Polynya. Atmosphere-Ocean.

[20] Dumont D, Gratton Y, Arbetter T (2009). Modeling the Dynamics of the North Water Polynya Ice Bridge. Journal of Physical Oceanography.

[21] Nutt DC (1969). The North Water of Baffin Bay. Polar Notes.

[22] Muench, R. *The physical oceanography of the northern Baffin Bay region*. (University Microfilms International, 1977).

[23] Ito H (1982). Wind Through a Channel—Surface Wind Measurements in Smith Sound and Jones Sound in Northern Baffin Bay. Journal of Applied Meteorology.

[24] Melling H, Gratton Y, Ingram G (2001). Ocean circulation within the North Water polynya of Baffin Bay. Atmosphere-Ocean.

[25] Vincent, R. & Marsden, R. A Study of Tidal Influences in the North Water Polynya using Short Time Span Satellite Imagery. *ARCTIC* **61** (2009).

[26] Steffen K (1985). Warm water cells in the North Water, Northern Baffin Bay during winter. Journal of Geophysical Research.

[27] Steffen K (1986). Ice Conditions of an Arctic Polynya: North Water in Winter. Journal of Glaciology.

[28] Preußer A, Heinemann G, Willmes S, Paul S (2015). Multi-Decadal Variability of Polynya Characteristics and Ice Production in the North Water Polynya by Means of Passive Microwave and Thermal Infrared Satellite Imagery. Remote Sensing.

[29] Dunphy, M., Dupont, F., Hannah, C. & Geenberg, D. *Validation of a modelling system for tides in the Canadian Arctic Archipelago* (2005).

[30] Kwok, R. Variability of Nares Strait ice flux. *Geophysical Research Letters* **32** (2005).

[31] Kwok, R., Toudal Pedersen, L., Gudmandsen, P. & Pang, S. Large sea ice outflow into the Nares Strait in 2007. *Geophysical Research Letters* **37** (2010).

[32] Ryan PA, Münchow A (2017). Sea ice draft observations in Nares Strait from 2003 to 2012. Journal of Geophysical Research: Oceans.

[33] Moore GWK, Schweiger A, Zhang J, Steele M (2019). Spatiotemporal variability of sea ice in the arctic’s last ice area. Geophysical Research Letters.

[34] Hastrup K, Andersen A, Grønnow B, Heide-Jørgensen M (2018). Life around the North Water ecosystem: Natural and social drivers of change over a millennium. Ambio.

[35] Hastrup, K. *Waterworlds* (BERGHAHN Books, 2015).

[36] Markham, C. & Baffin, W. *The voyages of William Baffin* (Routledge, 2016).

[37] Hayes, I. *The open Polar Sea: a narrative of a voyage of discovery towards the North Pole* (Hurd and Houghton, 1867).

[38] Dunbar M, Dunbar M (1972). The History of the North Water. Proceedings of the Royal Society of Edinburgh. Section B. Biology,.

[39] Dunbar, M. The Geographical Position of the North Water. *ARCTIC* **22** (1969).

[40] Vincent R (2013). The 2009 North Water Anomaly. Remote Sensing Letters.

[41] Liang, S. (ed), *Comprehensive Remote Sensing*. (1st ed. Elsevier, 2017).

[42] Orvig, S. *Climates of the Polar Regions* (Elsevier Pub. Co., 1970).

[43] Herman G, Goody R (1976). Formation and Persistence of Summertime Arctic Stratus Clouds. Journal of the Atmospheric Sciences.

[44] Heinemann G (2018). An Aircraft-Based Study of Strong Gap Flows in Nares Strait, Greenland. Monthly Weather Review.

[45] Moore GWK, Våge K (2018). Impact of model resolution on the representation of the air-sea interaction associated with the North Water Polynya. Quarterly Journal of the Royal Meteorological Society.

[46] Dunbar, M. Fall Ice Drift in Nares Strait, as Observed by Sideways-Looking Airborne Radar. *ARCTIC* **32** (1979).

[47] Vincent R, Marsden R, McDonald A (2001). Short time‐span ice tracking using sequential AVHRR imagery. Atmosphere-Ocean.

[48] Barber DG (2018). Increasing Mobility of High Arctic Sea Ice Increases Marine Hazards Off the East Coast of Newfoundland. Geophysical Research Letters.

[49] Korosov AA (2018). A new tracking algorithm for sea ice age distribution estimation. The Cryosphere.

[50] Kwok R (2009). Outflow of Arctic Ocean Sea Ice into the Greenland and Barents Seas: 1979–2007. Journal of Climate.

[51] Howell, S. E. L. & Brady, M. The Dynamic Response of Sea Ice to Warming in the Canadian Arctic Archipelago. *Geophysical Research Letters* (2019).

[52] Moore GWK, Mcneil K (2018). The Early Collapse of the 2017 Lincoln Sea Ice Arch in Response to Anomalous Sea Ice and Wind Forcing. Geophysical Research Letters.

[53] Serreze M, Francis J (2006). The Arctic Amplification Debate. Climatic Change.

[54] Solomon, S. *Climate change 2007* (Cambridge University Press, 2008).

[55] Lindsay R, Zhang J (2005). The Thinning of Arctic Sea Ice, 1988–r2003: Have We Passed a Tipping Point?. Journal of Climate.

[56] Screen J, Simmonds I (2010). The central role of diminishing sea ice in recent Arctic temperature amplification. Nature.

